# Rethinking verbal fluency ‐ development of an automated toolkit for analysing traditional and executive task variants

**DOI:** 10.1002/alz70857_102647

**Published:** 2025-12-24

**Authors:** Alice Stanton, Claire L Lancaster, Naji Tabet, Jennifer M Rusted

**Affiliations:** ^1^ University of Sussex, Brighton, East Sussex, United Kingdom; ^2^ Brighton & Sussex Medical School, Brighton, United Kingdom; ^3^ Brighton & Sussex Medical School, Brighton, East Sussex, United Kingdom; ^4^ University of Sussex, Brighton, United Kingdom

## Abstract

**Background:**

Verbal fluency tasks (VFTs) are valuable for detecting early neurodegenerative disease. Automated VFT scoring methods may enhance dementia diagnosis by extracting metrics of lexical access, semantic clustering, production timings, and acoustic speech features. This research presents an automated dashboard which quantifies and visualizes verbal fluency features, designed to enhance the accessibility and feasibility of using fine‐gained performance metrics. Data from adults across the lifespan showcases this tool's sensitivity to age effects from earlier in the lifespan.

**Methods:**

Seventy‐five cognitively healthy adults (aged 20‐80 years) completed a VFT battery, with data collection ongoing (target *n* = 120). Tasks include traditional and novel variants of the verbal fluency paradigm, with category switching, response inhibition, semantic association, and multi‐tasking differentially challenged across tests. Audio recordings were processed by the dashboard, outputting metrics including semantic clustering, switch reaction time costs, and pause duration for analyses.

**Results:**

The automated pipeline confirmed well‐established age‐related effects on conventional verbal fluency performance, with young adults producing significantly more responses compared to mid‐age and older adults (F(2,60) = 8.92, p < .001, η^2^_p_ = .229). While mid‐age adults demonstrated intermediate performance between young and older adults on traditional VFTs, automated metrics revealed additional cognitive changes. Specifically, the switch task showed steeper performance declines in the initial 0‐15 seconds for mid‐age adults compared to young adults (Age × Time interaction: F(14,420) = 2.47, p = .012, η^2^_p_ = .076). Additionally, mid‐age adults exhibited greater dual‐task costs (VFT: F(1,60) = 123.76, p < .001) and dissociation costs (M = 1.80s) compared to younger adults (M = 1.39s).

**Conclusions:**

Results provide early support for this automated verbal fluency dashboard in detecting subtle cognitive outcomes, particularly with varying executive demands. By establishing normative data across age groups and integrating findings into an interactive clinical dashboard, this research advances development of sensitive diagnostic tools. Future work will incorporate APOE genotype and clinical populations to validate the dashboard's utility in enhancing dementia diagnostics and care, while establishing clinical feasibility and acceptability.

